# A Case Report of Cannabis Abuse: A Surprising Etiology of Elevated Troponin

**DOI:** 10.7759/cureus.33423

**Published:** 2023-01-05

**Authors:** Nway Nway, Sagar Pandey, Tutul Chowdhury, Malavika Shankar, Kalpana Panigrahi

**Affiliations:** 1 Internal Medicine, Interfaith Medical Center, New York, USA; 2 Internal Medicine, Interfaith Medical Center, Brooklyn, USA; 3 Internal Medicine, One Brooklyn Health System, Brooklyn, USA

**Keywords:** substance-abuse”, : acute coronary syndrome, high troponin, cannabis use, cardiac troponin

## Abstract

Cannabis is the most commonly used additive drug after alcohol and tobacco. There has been literature proving the relationship between cannabis use and elevated troponin from myocardial infarction, with many mechanisms explaining them. However, limited data are available on elevated troponin due to cannabis-induced high myocardial oxygen demand due to vasospasm. We present a case of a 21-year-old female presenting with chest pain after cannabis abuse. She exhibited a steep rise in troponin with a normal electrocardiogram (EKG). She refused a coronary angiogram, but a bedside echocardiogram showed no wall motion abnormality. Therefore, the dramatic rise of troponin levels with the chest pain and the resolution of the symptoms were most likely explained by demand ischemia via the mechanism of reversible vasospasm.

## Introduction

Cannabis is one of the most widely used illicit substances, which severely impacts cardiovascular systems [1, 2]. Marijuana acts on the cannabinoid receptors with dose-dependent effects on the cardiovascular system, such as tachycardia, hypertension, cardiac arrhythmias, atrioventricular (AV) block, atrial fibrillation, ventricular fibrillation, and asystole. Despite these reported side effects, the use of marijuana has still been on the rise. Cannabis can cause elevated heart rate and blood pressure immediately after use from stimulation of the sympathetic nervous system. The recreational use of marijuana has caused an increase in the number of serious cardiovascular complications across the world. We present a case where a cannabis abuser presents chest pain with drastic elevation in troponin.

## Case presentation

A 21-year-old female patient with a past medical history of asthma presented with acute onset of chest pain. She localized the pain over the chest and epigastric area. It was moderate to severe, non-radiating and non-exertional pain relieved by sitting upright and leaning forward. It was associated with multiple episodes of nausea and vomiting with yellow-colored non-bloody vomitus containing ingested food particles. She denied any history of fever, cough, shortness of breath, palpitation, abdominal pain, or lower urinary tract symptoms. She also denied any illicit drug abuse on presentation.

**Figure 1:**
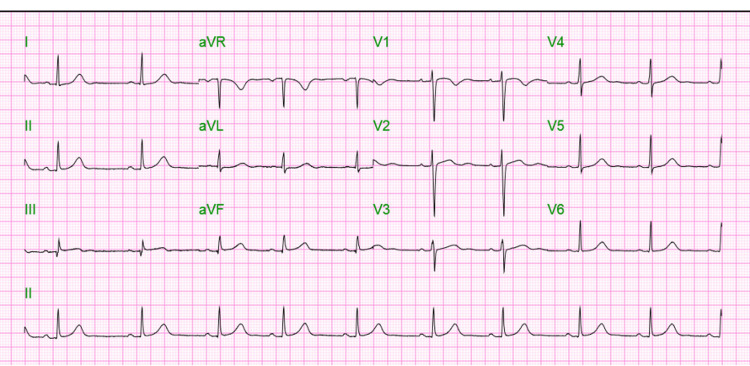
Electrocardiogram (EKG) at presentation showing sinus rhythm

On examination, blood pressure was 147/89 mmHg with a pulse rate of 64 per minute, respiratory rate of 18 per minute, a temperature of 36.9 degrees C, and saturation of 99% on room air. Auscultation of the chest revealed bilateral equal air entry with no added breath sound and normal heart sounds. The abdominal examination was uneventful, and there was no edema in the extremities. The initial electrocardiogram (EKG) showed normal sinus rhythm with a heart rate of 73 beats per minute, PR interval of 172 ms, and QRS duration of 90 ms with QTc of 453 ms (Figure 1).

**Figure 2:**
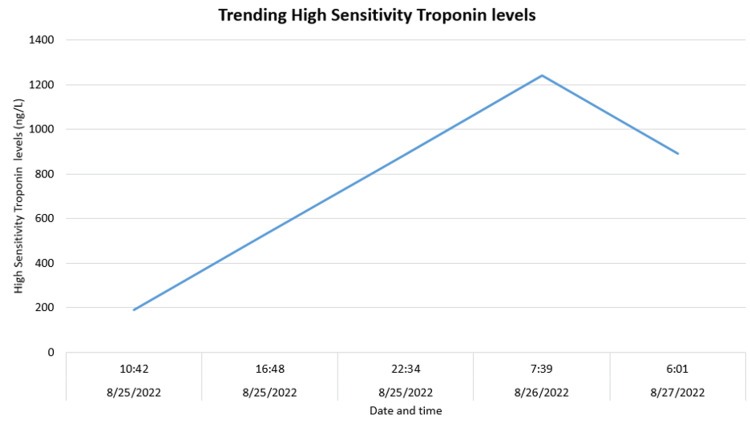
Graphical representation of troponin levels trend

The initial laboratory tests showed white cell counts of 5800/mm3, hemoglobin 13.3 g/dl, and hematocrit 42%. The d-dimer was high at 663 ng/ml. The urine toxicology screen was positive for cannabinoids and opioids. However, urine toxicology positive for opioids can be justifiable as the urine sample was collected after she received morphine in the emergency department for pain, and the urine sample was collected subsequently. Furthermore, high sensitivity troponin at the time of admission was 190.9 ng/ml, which later trended to a maximum value of 1234.2 ng/ml on day two of admission. Figure 2 depicts the graphical representation of troponin levels on the day of admission.

**Figure 3:**
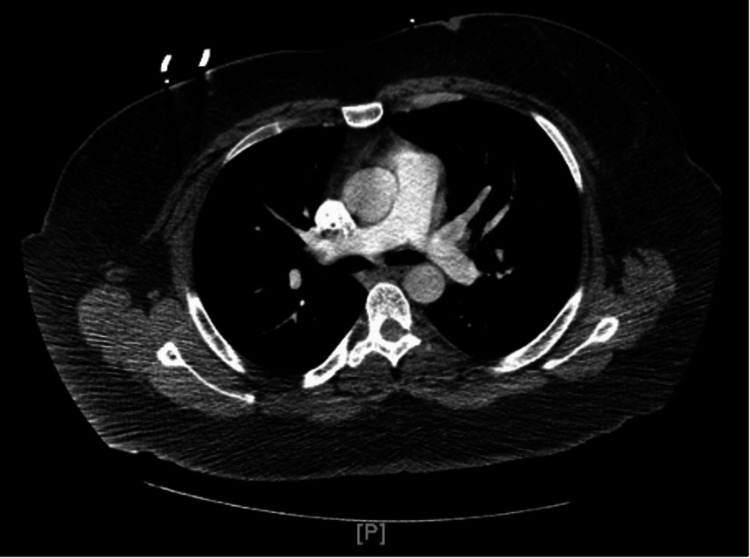
The Computed Tomography Angiogram (CTA) scan of the chest showed no evidence of pulmonary embolism

The computed tomography angiogram (CTA) of the chest was ordered to rule out pulmonary embolism, which showed no definite evidence of pulmonary embolism with no acute pleural or parenchymal disease (Figure 3).

Bedside echocardiography showed an ejection fraction of 60% with no regional wall motion abnormalities with no signs of effusion. We offered the patient coronary angiography to rule out any coronary artery disease, but she refused the procedure after discussing its risks and benefits. 

**Figure 4:**
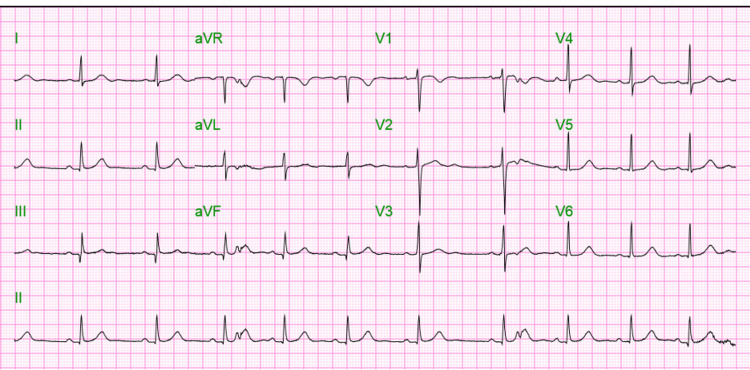
Electrocardiogram (EKG) on Day 4 showing Normal sinus rhythm.

She was managed with aspirin 324 mg stat, atorvastatin 80 mg, intravenous morphine sulfate 4 mg, intravenous ondansetron, and sublingual nitroglycerine 0.4 mg. She was admitted to the telemetry unit for continuous cardiac monitoring. Her symptoms resolved eventually, with complete recovery on day four of admission. She received continued treatment with aspirin 81 mg daily and atorvastatin 40 mg daily. Repeat EKG (Figure 4) showed normal sinus rhythm with a heart rate of 64 bpm, PR interval of 170 ms, QRS interval of 90 ms, and QTc interval of 455 ms. 

The patient was discharged on day four of admission with the discharge diagnosis of chest pain with elevated troponin, likely due to stress demand secondary to cannabis use. Aspirin 81 mg chewable tablets and atorvastatin 40 mg tablets were continued as discharge medication with advice to follow up in the cardiology clinic in one week.

## Discussion

Cannabis is one of the widely used psychoactive substances with euphoric effects mainly via the active compound tetrahydrocannabinol (THC) and inflammatory effects via cannabidiol (CBD) [1, 2]. Its effects are mediated via CB1 and CB2 receptors, distributed over the central nervous system, cardiovascular system, and immune cells [3].

In this case report, we discussed the elevated troponin levels in a 21-year-old nonsmoker female with no history of type 2 diabetes mellitus, hypertension, hyperlipidemia, or chronic kidney disease. However, the patient refused percutaneous coronary intervention to visualize the coronary arteries, a normal EKG in a patient without any established cardiovascular risk factors for coronary artery disease (CAD) points toward a low pretest probability of CAD as a cause of troponin elevation. On the other hand, elevated troponin, likely due to stress demand due to cannabis use, is a genuine possibility. Jouanjus et al. and Mittleman et al., in large-scale retrospective studies, reported the incidence of cardiovascular events as 2.6 per 1000 in regular cannabis users, along with an annual acute cardiovascular event risk of 1.5 to 3% per year [4, 5]. There has been literature proving a cannabis-induced increase in myocardial oxygen demand. The proposed mechanisms for the increase in myocardial oxygen demand are from tachycardia due to the activation of the CB1 receptor-mediated sympathetic nervous system, the inhibition of the cardiac parasympathetic nervous system, and reflex tachycardia from cannabis-induced vasodilation [6-8].

Furthermore, an increase in myocardial oxygen demand is also attributed to reduced oxygen delivery due to carboxyhemoglobin accumulation in people smoking cannabis [9]. In addition, cannabis induced endothelial dysfunction and platelet aggregation could also lead to compromised myocardial vasculature impairing oxygen delivery to myocardial tissues [10-12]. In our case, the circumstantial evidence of having no risk factors for acute coronary syndrome, the rise of troponin with the symptoms, and the resolution of the symptoms afterward can be proved by the increased myocardial oxygen demand likely from vasospasm.

## Conclusions

This case report serves as one of the rare presentations of cannabis-induced elevated troponin, which trended down after cessation of cannabis intake. It is imperative to consider cannabis-induced elevated troponin for the workup of chest pain in cannabis users after ruling out the most common causes. Hence, it opens a new chapter of discussion for the lesser explored area in the management approach, like aspirin and anticoagulation and the long-term prognosis of cannabis-induced elevated troponin.
